# Octreotide Subcutaneous Depot for Acromegaly: A Randomized, Double-blind, Placebo-controlled Phase 3 Trial, ACROINNOVA 1

**DOI:** 10.1210/clinem/dgae707

**Published:** 2024-10-08

**Authors:** Diego Ferone, Pamela Freda, Laurence Katznelson, Federico Gatto, Pinar Kadioğlu, Pietro Maffei, Jochen Seufert, Julie M Silverstein, Joanna L Spencer-Segal, Elena Isaeva, Alexander Dreval, Maria Harrie, Agneta Svedberg, Fredrik Tiberg

**Affiliations:** Endocrinology Unit, Department of Internal Medicine, IRCCS Ospedale Policlinico San Martino, 16131 Genova, Italy; Department of Medicine, Vagelos College of Physicians & Surgeons, Columbia University, New York, NY 10032, USA; Departments of Medicine and Neurosurgery, Stanford University, Stanford, CA 94305, USA; Endocrinology Unit, Department of Internal Medicine, IRCCS Ospedale Policlinico San Martino, 16131 Genova, Italy; Division of Endocrinology-Metabolism and Diabetes, Department of Internal Medicine, Istanbul University-Cerrahpasa, 34303 Istanbul, Turkey; Department of Medicine, Padua University Hospital, 35128 Padua, Italy; Division of Endocrinology and Diabetology, Department of Medicine II, Medical Faculty, University of Freiburg, 79110 Freiburg, Germany; Division of Endocrinology, Metabolism and Lipid Research, Washington University School of Medicine, St. Louis, MO 63110, USA; Department of Internal Medicine and Michigan Neuroscience Institute, University of Michigan, Ann Arbor, MI 48109, USA; Interregional Clinical Diagnostic Center, Kazan 420101, Russia; Endocrinology Department, Moscow Regional Research Clinical Institute, Moscow 117292, Russia; Camurus AB, 223 62 Lund, Sweden; Camurus AB, 223 62 Lund, Sweden; Camurus AB, 223 62 Lund, Sweden

**Keywords:** acromegaly, octreotide, CAM2029, FluidCrystal, somatostatin receptor ligands, randomized controlled trial

## Abstract

**Context:**

Acromegaly, characterized by excessive GH and insulin-like growth factor-1 (IGF-1), impacts quality of life (QoL) and mortality. Standard of care (SoC; octreotide long-acting repeatable or lanreotide autogel) treatment typically requires healthcare provider administration. CAM2029, a novel subcutaneous octreotide depot with increased bioavailability using FluidCrystal technology, enables self-administration and room-temperature storage.

**Objective:**

Assess superiority of CAM2029 vs placebo for biochemical control in patients with controlled acromegaly.

**Design:**

24-week, multinational, randomized, double-blind, phase 3 trial (NCT04076462).

**Setting:**

45 sites; 10 countries.

**Patients:**

72 patients on SoC with biochemical control at screening [IGF-1 ≤upper limit of normal (ULN); mean GH <2.5 μg/L].

**Interventions:**

Patients were randomized 2:1 to once-monthly CAM2029 (n = 48) or placebo (n = 24).

**Main Outcome Measures:**

The primary endpoint was proportion of patients with IGF-1 ≤ULN (week 22/24 mean), with dose-reduced patients classified as nonresponders; first key secondary endpoint was the same, including dose-reduced responders. The second key secondary endpoint was proportion of patients with IGF-1 ≤ULN (week 22/24) and mean GH <2.5 μg/L (week 24).

**Results:**

At week 22/24 (intention-to-treat analysis), CAM2029-treated patients demonstrated superior response rates vs placebo for IGF-1 (72.2% vs 37.5%; risk difference: 34.6, 95% confidence interval: 11.3, 57.9; *P* = .0018) and combined IGF-1/GH (70.0% vs 37.5%; *P* = .0035). CAM2029-treated patients had well-controlled symptoms, improved QoL, and treatment satisfaction vs placebo and baseline. CAM2029 was well tolerated; safety was consistent with SoC.

**Conclusion:**

CAM2029 provides a convenient and effective treatment option for acromegaly, with superior biochemical control vs placebo. Symptom control, QoL, and satisfaction were improved from baseline SoC.

**Clinical Trial Registration:**

NCT04076462 (ClinicalTrials.gov).

Acromegaly is a rare systemic endocrine disorder, typically caused by a pituitary adenoma, occurring in 5.9/100 000 individuals, globally (1). The condition is characterized by excessive GH release and elevated insulin-like growth factor-1 (IGF-1) (2, 3), the latter being the most reliable disease activity biomarker (4). In patients without biochemical control of their condition, resulting phenotypic alterations and comorbidities reduce quality of life (QoL) and can increase morbidity and mortality (1-6).

Although surgery is first-line therapy in the vast majority, many patients require further treatments to achieve GH and IGF-1 control (4, 6, 7). Injectable first-generation somatostatin receptor ligands (SRLs) octreotide and lanreotide reduce GH and IGF-1 production and are standard of care (SoC) first-line pharmacological treatments (8, 9). However, these treatments have complex storage procedures and typically require administration by a trained healthcare provider, increasing the treatment burden (10-14). While oral octreotide is available, it requires twice-daily fasted dosing and has very low bioavailability, which varies according to food intake (15, 16).

Often, even in those who experience control of IGF-1 with SRL treatments, patients continue to report persistent disease symptoms (3, 17-19). Furthermore, patients frequently report poor QoL and low treatment satisfaction with existing SRLs due to the persistent symptoms and treatment burden (20). This indicates that a convenient treatment option for acromegaly that is not only effective for both biochemical and symptom control but also optimizes patients’ outcomes, including QoL, would be highly valuable for patients (21).

With the goal of providing high levels of disease control while crucially lowering treatment burden and improving patients’ QoL, a novel formulation of octreotide using proprietary FluidCrystal technology was developed. This octreotide subcutaneous depot (CAM2029) has ∼5-fold higher bioavailability than octreotide long-acting repeatable (LAR) (22, 23), is stored at room temperature, and is administered using prefilled auto-injection pens suitable for self-injection. CAM2029 also utilizes a thinner, higher gauge needle (22G) than SoC (18G). The solution transforms in situ to a liquid-crystalline gel and encapsulated octreotide is released as the depot matrix biodegrades (24).

The primary aim of this study was to assess the superiority of CAM2029 vs placebo in maintaining biochemical control in patients with controlled acromegaly on stable SoC treatment. Further objectives included assessment of changes in symptom severity, QoL, and treatment satisfaction, in addition to the evaluation of safety.

## Materials and Methods

### Study Design

This was a multinational, randomized, double-blind, phase 3 trial assessing the efficacy and safety of CAM2029 vs placebo in patients with acromegaly. The trial was conducted at 45 sites (including hospitals, clinics, and clinical research centers) across 10 countries (Russia, Turkey, Italy, Spain, Germany, United Kingdom, Hungary, Greece, Poland, United States), with patients randomized at 33 sites. The trial consisted of an ≤8-week screening phase and a randomized, 24-week, double-blind treatment phase. The primary objective was to assess the superiority of CAM2029 vs placebo for biochemical control in patients with controlled acromegaly on first-generation SRLs. The use of placebo for the control arm was a regulatory requirement. The trial was conducted in accordance with the Declaration of Helsinki; country-specific ethical approval was granted.

### Patients

Eligible patients (≥18 years) had confirmed acromegaly, had IGF-1 ≤upper limit of normal (ULN; adjusted for both sex and age at screening), had mean GH <2.5 μg/L (5 samples taken at 30-minute intervals), and were on stable once-monthly octreotide LAR (10/20/30/40 mg) or lanreotide autogel (ATG; 60/90/120 mg) for ≥3 months. Patients who had received pasireotide within 6 months, pegvisomant or dopamine agonists within 3 months, and other investigational agents within 30 days or 5 half-lives (whichever was longer) were excluded. Patients with prior pituitary surgery were only eligible if their surgery was performed >6 months prior to screening and had IGF-1 >ULN based on a measurement performed at least 3 months after the surgery. Sex was self-reported (male/female) by participants during screening. Patients provided written informed consent.

### Randomization and Masking

Eligible patients were randomized 2:1 to receive CAM2029 or placebo once monthly during the 24-week double-blind treatment phase (Supplementary Fig. S1) (25). Randomization was performed using an interactive web response system and stratified by prior treatment (octreotide LAR or lanreotide ATG). Patients, investigators, and the trial team remained blinded to treatment and IGF-1/GH results. To maintain blinding, CAM2029 and placebo treatments were identical in appearance, volume, and viscosity of the solutions.

### Procedures

Based on previous studies, the CAM2029 starting dose was 20 mg (1.0 mL prefilled syringes) regardless of prior treatment (22, 26). The first dose [CAM2029 or placebo (also 1.0 mL prefilled syringes)] was administered on day 1 of the treatment phase, 4 weeks (±3 days) after the last dose of octreotide LAR or lanreotide ATG and provided once monthly (±7 days) thereafter.

Treatment was administered at the trial site, in the morning (before noon), and as close as possible to the time of day that the first dose was received, irrespective of the number of preadministration assessments conducted during the visit. The trial was designed for participants to receive the first dose of CAM2029 in the same timeframe as they would otherwise have received their next dose of SoC treatment, in alignment with similar trials (27). Treatments were self-administered or administered by a partner or by trial personnel as subcutaneous injections in the abdomen or thigh. Doses could be down-titrated to 10 mg CAM2029 (0.5 mL), or 0.5 mL placebo, at any time based on safety and tolerability. Patients experiencing worsening acromegaly signs/symptoms could be switched to SoC if IGF-1, monitored by an independent reader, was ≥1.3 × ULN at 2 consecutive visits (including any unscheduled visits). All normal range and ULN IGF-1 assessments were adjusted for both sex and age at screening.

Patients attended an end-of-trial visit at week 24 or 4 weeks after the last dose in case of premature withdrawal. Patients completing the trial could enroll in a long-term, open-label safety trial of CAM2029 (ACROINNOVA 2; NCT04125836). The end of the trial was defined as the week 24 visit for patients who subsequently enrolled in the open-label trial. The end of the trial for all other patients was defined as the last protocol-specified contact with that patient (including follow-up of adverse events).

Blood samples for measuring IGF-1 and octreotide were collected predose on day 1 (ie, baseline, but for IGF-1 the mean of the screening measurement 2 weeks before day 1 and predose measurement on day 1 was used as the baseline value) and predose at weeks 4, 8, 12, 16, and 20. At week 20, blood samples were also collected at 2 ± 1, 5 ± 1, 8 ± 1, 24 ± 4, and 96 ± 24 hours postdose. Then, samples were taken at week 22 and 24 following final dose at week 20 (or end of trial if withdrawn). The IGF-1 concentrations in serum samples were analyzed in a central laboratory using a validated assay [IDS-iSYS IGF-1 (calibrated against WHO IS 02/254); Immunodiagnostic Systems], with a reported intra-assay coefficient of variation of 1.3% to 3.7% and interassay coefficient of variation of 3.4% to 8.7%, which has been used in other recent phase 3 trials (27-29). A mean of 2 measurements was used, as is commonly used in phase 3 acromegaly trials (27, 29, 30).

For GH measurement, 5 samples were obtained predose over 2 hours on day 1 and week 24, and random samples were obtained at weeks 4, 8, 12, 16, and 20 (predose) and week 22. GH concentration was analyzed in a central laboratory using a validated assay [IDS-iSYS hGH (calibrated against WHO IS 98/574); Immunodiagnostic Systems).

Sample collection was conducted at a similar time of day (morning, before noon) during the screening and treatment phases.

### Efficacy Outcomes

Efficacy analyses were based on the intention-to-treat (ITT) analysis set, comprising all randomized patients. The primary endpoint was the proportion of patients with mean IGF-1 ≤ULN at week 22 and 24 (mean of the 2 measurements), where, in addition to patients with IGF-1 >ULN, patients with treatment discontinuation, rescue medication, and reduced dose were classified as nonresponders. The first key secondary endpoint was the proportion of patients with mean IGF-1 ≤ULN (week 22/24 mean), with dose-reduced patients achieving IGF-1 ≤ULN classified as responders. The second key secondary endpoint was the proportion of patients with both mean IGF-1 ≤ULN (week 22/24 mean) and mean GH <2.5 μg/L (week 24).

The Acromegaly Index of Severity (AIS) (31) was completed by the investigator (with the patient), predose at each visit from baseline. Patients completed the Acromegaly QoL questionnaire (AcroQoL) (32), EQ-5D-5L (33), and Treatment Satisfaction Questionnaire for Medication (TSQM) v1.4 (34) predose at baseline (day 1) and at week 24/end of trial, alongside a Patient Satisfaction Scale (PSS) at week 24. Patients who self-administered treatment completed the Self-Injection Assessment Questionnaire (SIAQ) v2.0 PRE module before first self-administration and POST module after first self-administration and at week 20 (35). The interpretations of scores are described in Supplementary Material S1 (25).

### Safety Outcomes

Safety analyses included all patients administered ≥1 dose of CAM2029 or placebo. Safety was evaluated throughout the trial; adverse events (AEs) were coded using the Medical Dictionary for Regulatory Activities v25.0 and graded using the National Cancer Institute Common Terminology Criteria for Adverse Events v5.0 (Grades 1-3) (36); AEs considered life-threatening or resulting in death were not graded but were considered serious AEs (SAEs). AEs were followed up until final outcome or end of trial participation, whichever came first. Incidence of corrected QT interval (corrected for heart rate using Fridericia's cube root formula) >450 ms is reported.

### Statistical Analysis

The trial was designed to have 90% power to detect a treatment difference for the primary endpoint. The proportion of patients with biochemical control at week 22/24 was assumed to be 80% for the CAM2029 arm and 40% for the placebo arm, which was based on input from clinical experts, findings from other trials, and the trial duration (37). The 6-month trial duration was selected to avoid exposing patients to placebo treatment longer than necessary. Therefore, 78 patients, randomized 2:1 to CAM2029 or placebo, would be sufficient to detect a significant between-arm difference, based on the chi-squared test (with continuity correction).

Biochemical control evaluations (primary and key secondary endpoints) were conducted in a hierarchical, closed testing procedure. All normal range and ULN IGF-1 assessments were adjusted for sex and age at screening. A comparison was eligible for superiority testing only if all previous comparisons established superiority at the one-sided significance level of *P* < .025. For all hierarchical variables, a common risk difference test using Mantel-Haenszel stratum weights was used, stratified by prior treatment (octreotide LAR or lanreotide ATG).

For other efficacy endpoints, estimates of least squares (LS) means for change from baseline were calculated based on analyses of covariance and mean differences between arms were estimated. Time to loss of response, defined as the earliest time when 2 consecutive IGF-1 measurements were >ULN, was analyzed using a Cox proportional hazards model with treatment as the factor and baseline IGF-1 as a covariate, stratified by prior treatment. The estimated time to loss of response in each treatment arm was demonstrated using the Kaplan-Meier method.

All analyses were performed using SAS® Software version 9.4. Handling of missing data is reported in Supplementary Material S2 (25). The trial was registered on ClinicalTrials.gov: NCT04076462.

## Results

**Figure 1.**
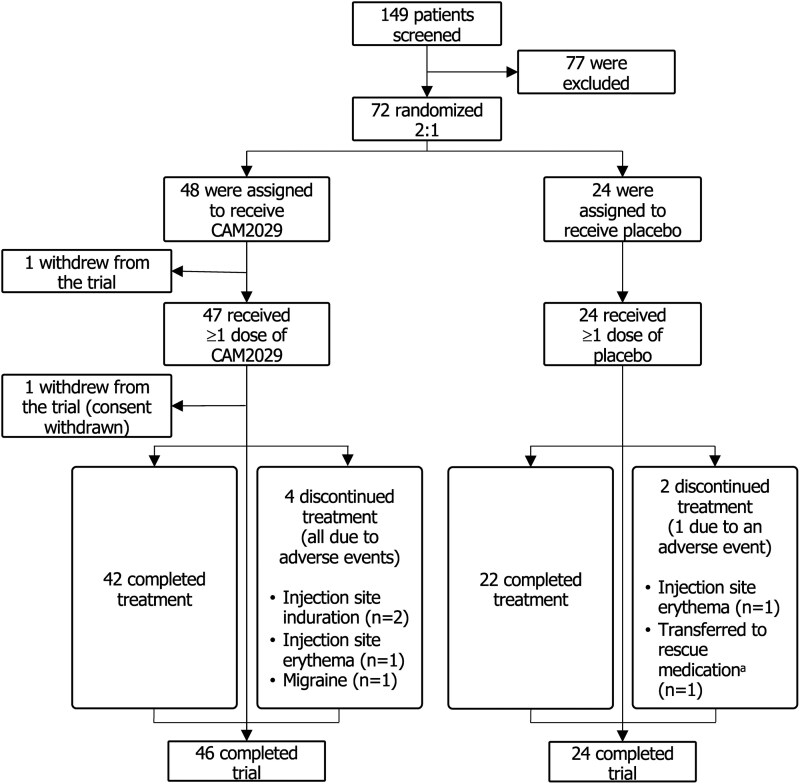
Patient flow diagram. ^a^Patients switched to rescue medication with SoC if they experienced worsening signs and symptoms of acromegaly together with an increase in IGF-1 to ≥1.3 × ULN at 2 consecutive visits. Abbreviations: IGF-1, insulin-like growth factor-1; SoC, standard of care; ULN, upper limit of normal.

Of 149 patients screened, 72 were eligible for participation in the trial and were randomized [CAM2029: 48; placebo: 24 (ITT analysis set)] between June 26, 2020, and November 17, 2022. In the CAM2029 arm, 2 patients withdrew (1 before receiving the first dose) and 46 completed the trial; 4 discontinued treatment due to AEs, and 42 completed treatment (Fig. 1). All 24 patients in the placebo arm completed the trial; 1 discontinued treatment due to an AE, 1 switched to rescue medication, and 22 completed treatment (Fig. 1).

Baseline characteristics were similar between treatment arms (Table 1). Mean time since diagnosis was >10 years in both arms; 39/72 patients (54.2%) and 33/72 patients (45.8%) were receiving treatment with octreotide LAR and lanreotide ATG, respectively. Mean (SD) time since first SRL treatment was similar between arms [CAM2029: 8.4 (5.7) years; placebo: 7.7 (5.6) years]. At baseline (mean of the second screening sample and day 1), 44/48 (91.7%) CAM2029-treated and 22/24 (91.7%) placebo-treated patients had IGF-1 ≤ULN. Predose on day 1 itself, 85.4% of patients in the CAM2029 arm and 91.7% in the placebo arm still had IGF-1 ≤ULN. Patients were representative of the wider acromegaly population with respect to age, sex, race, and ethnic group (Supplementary Table S1) (25).

**Table 1.** Patient baseline characteristics

Parameter	Statistics/category	CAM2029	Placebo
Patient demographics		n = 48	n = 24
Age (years)	Mean (SD)	57.0 (11.2)	52.0 (15.1)
	Range	29-79	20-82
	18-64, n (%)	34 (70.8)	19 (79.2)
	≥65, n (%)	14 (29.2)	5 (20.8)
Sex	Female, n (%)	28 (58.3)	12 (50.0)
	Male, n (%)	20 (41.7)	12 (50.0)
Weight (kg)	Mean (SD)	85.1 (17.6)*^a^*	87.1 (17.3)
Height (cm)	Mean (SD)	168.3 (11.0)*^a^*	171.5 (8.2)
BMI (kg/m^2^)	Mean (SD)	30.1 (5.6)*^a^*	29.7 (5.8)
**Acromegaly history**			
Time since diagnosis (years)	Mean (SD)	10.8 (6.8)*^a^*	13.0 (10.7)
Time since diagnosis (years)	<10, n (%)	23 (47.9)	11 (45.8)
	≥10-<20, n (%)	19 (39.6)	9 (37.5)
	≥20, n (%)	5 (10.4)	4 (16.7)
Pituitary surgery*^b^*	Yes, n (%)	42 (87.5)	21 (87.5)
	No, n (%)	6 (12.5)	3 (12.5)
Treatment at baseline	Octreotide LAR, n (%)	25 (52.1)	14 (58.3)
	Lanreotide ATG, n (%)	23 (47.9)	10 (41.7)
Time since first recorded SRL treatment (years)*^c^*	Mean (SD)	8.4 (5.7)*^a^*	7.7 (5.6)*^d^*
	Range	0.9-27.2	0.5-18.2

ITT analysis set.

Abbreviations: ATG, autogel; BMI, body mass index; ITT, intention-to-treat; LAR, long-acting repeatable; SRL, somatostatin receptor ligands.

^*a*^n = 47.

^*b*^Patients who had undergone pituitary surgery needed to have IGF-1 >ULN based on a measurement performed at least 3 months after the surgery to be eligible.

^*c*^Time since first recorded treatment with octreotide LAR or lanreotide ATG to first dose (day 1).

^*d*^n = 23.

**Figure 2.**
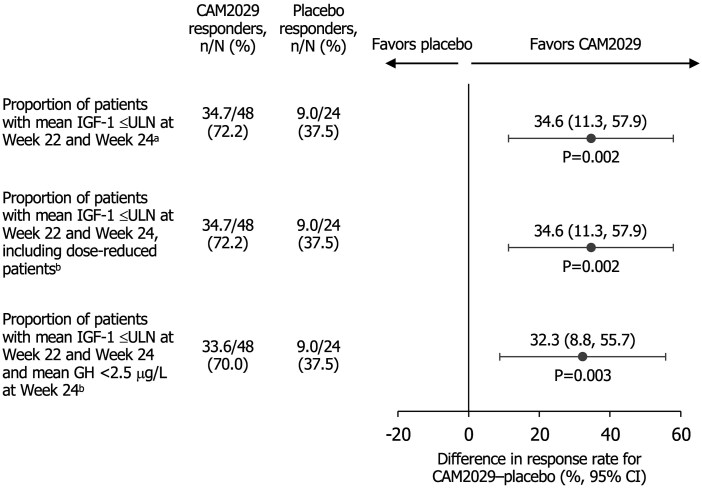
Biochemical control at week 22 and 24 (primary and key secondary efficacy endpoints). Proportion (percentages) of patients with biochemical response at the end of trial. ITT analysis set. ^a^Primary efficacy endpoint; ^b^Key secondary efficacy endpoint. *P*-values are one-sided. Combined estimates from 100 analyses based on imputed dataset. Patients with intercurrent events were regarded as nonresponders independently of their endpoint result; Mantel-Haenszel-type common difference in proportions across strata, stratified by prior treatment (octreotide LAR or lanreotide ATG). In the closed testing procedure, a comparison was eligible for superiority testing only if all previous comparisons, if any, had established superiority at the one-sided significance level of *P* < .025. Abbreviations: ATG, autogel; CI, confidence interval; IGF-1, insulin-like growth factor-1; ITT, intention-to-treat; LAR, long-acting repeatable; ULN, upper limit of normal.

The primary endpoint was met. At week 22/24, CAM2029-treated patients demonstrated a superior IGF-1 response rate (72.2%) vs placebo (37.5%). The estimated between-arm difference of 34.6% [95% confidence interval (CI): 11.3, 57.9] was statistically significant (*P* = .0018, one-sided); the odds ratio (CAM2029/placebo) was 4.31 (95% CI: 1.52, 12.27; Fig. 2). As no patient had a dose reduction, the primary and the first key secondary endpoint results were identical (Fig. 2). For the second key secondary endpoint, significantly more CAM2029-treated patients had both mean IGF-1 ≤ULN at week 22/24 and mean GH <2.5 μg/L at week 24 vs placebo-treated patients (70.0% vs 37.5%, respectively; *P* = .0035, one-sided; Fig. 2). Sensitivity and supportive analyses confirmed the main analyses’ conclusions (Supplementary Tables S2 and S3) (25).

**Figure 3:**
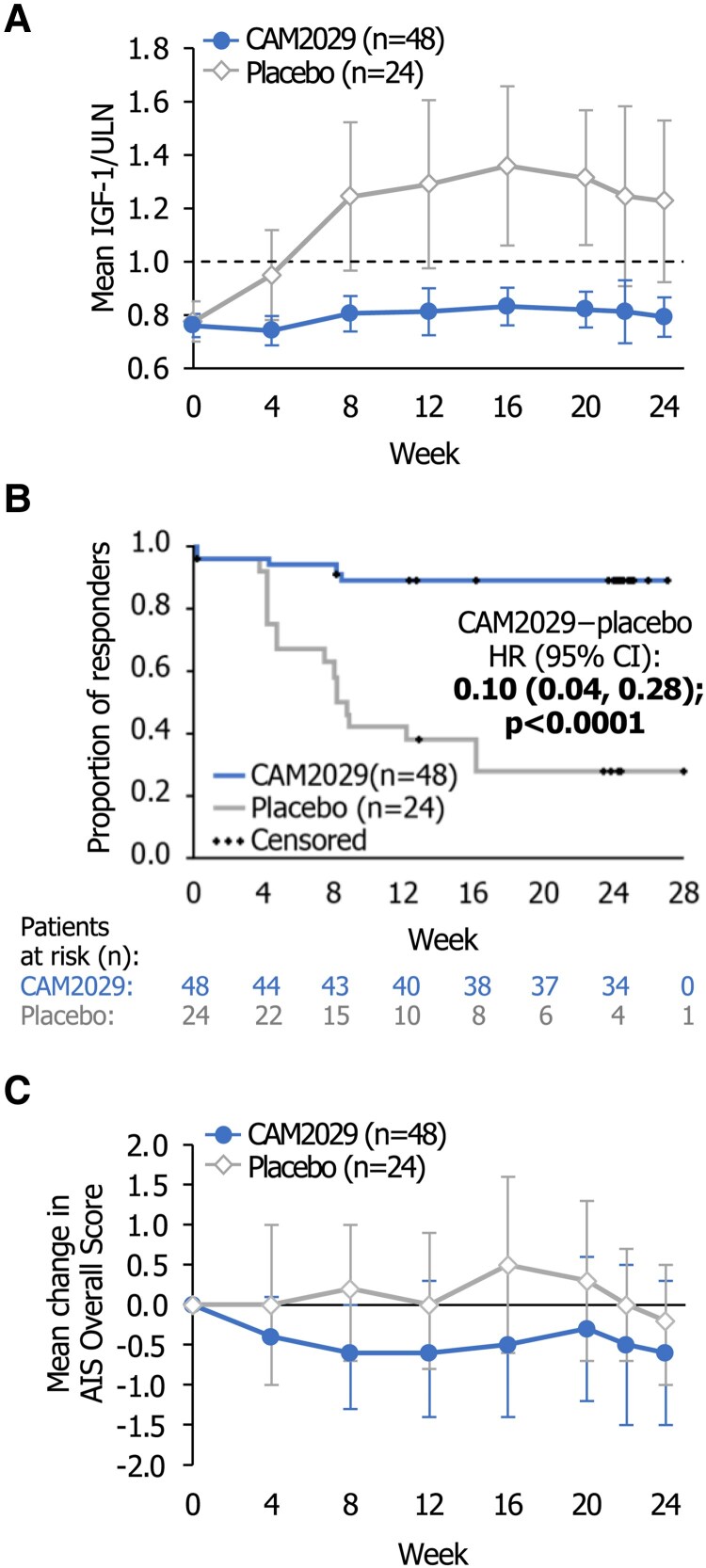
. Biochemical and symptom control over time. ITT analysis set. (A) Mean IGF-1 values (absolute values as a ratio to the ULN) for all patients in the 2 treatment arms over time; (B) Time to loss of IGF-1 response;^a^ (C) Mean change in AIS Overall Score from baseline over time.^b^ Error bars show 95% CIs. ^a^Time to loss of response was defined as the earliest time when 2 consecutive IGF-1 measurements were >ULN. For patients categorized as nonresponders at their first and second consecutive measurement, loss of response was reported as day 1. Responders still receiving treatment were censored at week 24. For patients who discontinued treatment or withdrew from the trial, IGF-1 values from the visit following their last injection were used, but any subsequent values were considered missing. Patients who discontinued treatment or withdrew without a confirmed loss of response were censored at their last available IGF-1 measurement; ^b^The AIS completed by the investigator (with the patient) predose at each visit. Abbreviations: AIS, Acromegaly Index of Severity; CI, confidence interval; IGF-1, insulin-like growth factor-1; ITT, intention-to-treat; ULN, upper limit of normal.

For CAM2029-treated patients, mean IGF-1 remained stable at ≤ULN throughout the trial (Fig. 3A), with a LS mean change from baseline of 0.04 (95% CI: −0.13, 0.20) in IGF-1/ULN at the mean of week 22/24. In the placebo arm, IGF-1/ULN increased to a mean of 1.24 IGF-1/ULN at week 8 and remained >ULN for the remainder of the trial, with a substantially higher LS mean change from baseline of 0.52 (95% CI: 0.31, 0.73) IGF-1/ULN at mean of week 22/24 vs CAM2029 [mean difference (CAM2029−placebo): −0.48; 95% CI: −0.75, −0.22]. Median time to loss of response was not reached in the CAM2029 arm and was 8.4 weeks in the placebo arm [hazard ratio (CAM2029−placebo): 0.10; 95% CI: 0.04, 0.28; Fig. 3B].

**Figure 4.**
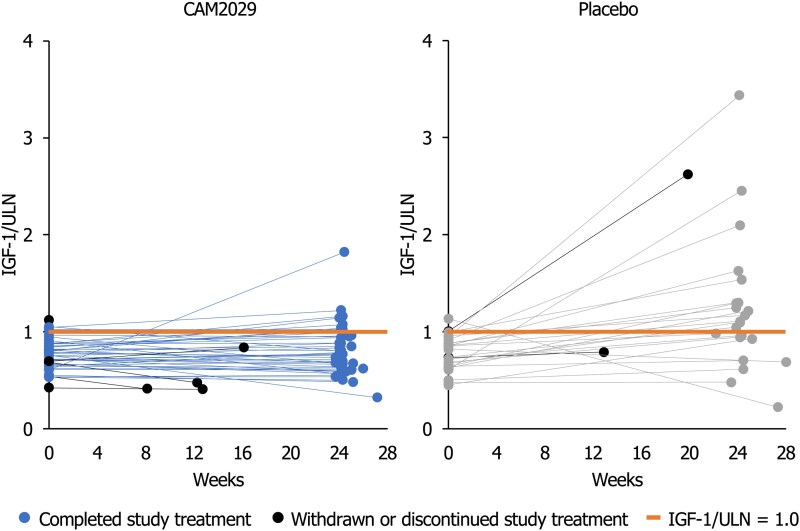
Individual IGF-1/ULN at baseline and at the end of trial. ITT analysis set. Individual IGF-1/ULN at baseline (mean of week −2 and day 1) and at the end of the trial (mean of week 22 and week 24) by actual time of last measurement (final visit was slightly later than week 24 for some patients, often due to COVID-19-related delays). For patients who withdrew or discontinued treatment, the last IGF-1 value before discontinuation was included. ^a^One patient in the CAM2029 arm had a high IGF-1 value at week 22 that subsequently dropped below ULN at week 24. Abbreviations: IGF-1, insulin-like growth factor-1; ITT, intention-to-treat; ULN, upper limit of normal.

Examining IGF-1/ULN data for individual patients, 8.3% of participants in each arm (CAM2029: 4/48; placebo: 2/24) had already lost biochemical control by baseline. Nevertheless, all but 1 patient in the CAM2029 arm had IGF-1 values below or slightly above the ULN at baseline and the end of the trial, while most patients in the placebo arm had IGF-1 values slightly or substantially above the ULN at the end of the trial after biochemical control was lost (Fig. 4). While 1 patient in the CAM2029 arm had a high IGF-1 value at week 22 that subsequently dropped below ULN at week 24, most CAM2029-treated responders did not lose biochemical control at the individual patient level, while most placebo-treated patients, including those counted as responders, had higher IGF-1 values at week 22/24 than baseline (Fig. 4). The difference in proportions of patients with IGF-1 ≤ULN between baseline and the mean of week 22/24 was −55.6% (95% CI: −77.8%, −33.4%) in placebo-treated patients, compared to −20.5% (95% CI: −33.7%, −7.3%) in CAM2029-treated patients. Among CAM2029-treated patients, it was notable that those who discontinued treatment due to AEs prior to week 22 and were regarded as nonresponders in the primary and secondary analyses had IGF-1 ≤ULN at their final assessment before switching back to their previous treatment for acromegaly.

Throughout the trial, mean GH remained <2.5 μg/L in both treatment arms and ≤1.0 μg/L in CAM2029-treated patients. In placebo-treated patients, mean GH exceeded 1.0 μg/L at week 4 [mean: 1.07 (95% CI: 0.43, 1.70)] and remained >1.0 μg/L thereafter (1.07-2.08 μg/L).

Clinical signs and symptoms of acromegaly assessed by the investigator using the AIS Overall Score were higher (more severe symptoms) at baseline, during stable treatment with SoC, in the CAM2029 arm [mean: 4.7 (95% CI: 3.7, 5.6)] than the placebo arm [mean: 2.4 (95% CI: 1.3, 3.4)]. The mean AIS Overall Score decreased below the SoC baseline value during treatment with CAM2029, indicating that symptoms were well controlled (Fig. 3C).

**Figure 5.**
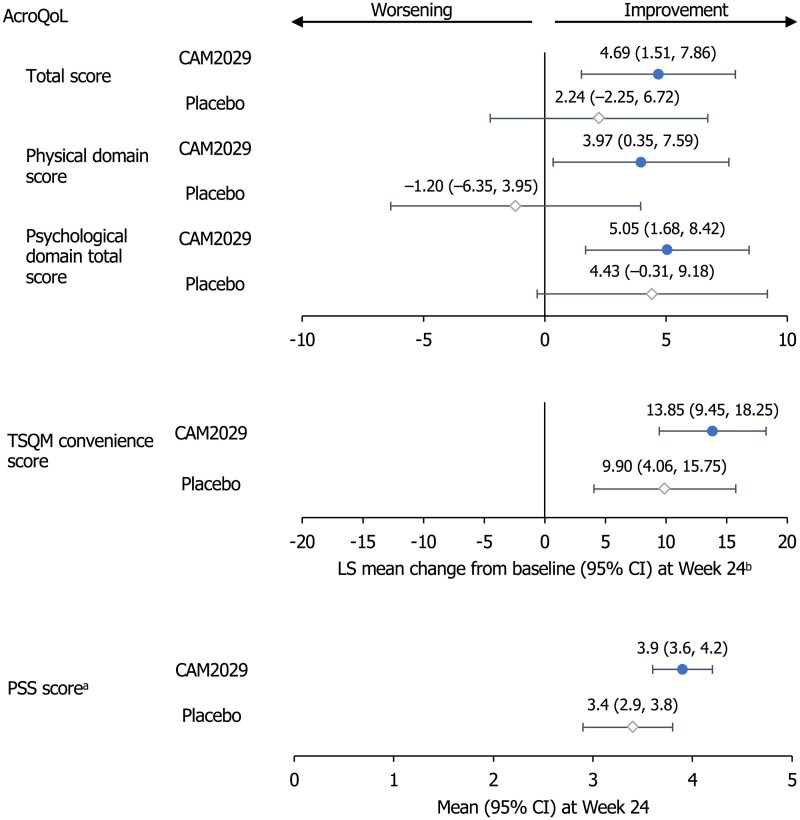
Key patient-reported outcomes. ITT analysis set. At baseline, patients were receiving SoC. Filled circles show results for the CAM2029 arm and open circles the placebo arm. ^a^Patients rated overall treatment experience compared to their prior treatment with octreotide LAR or lanreotide ATG from 1 “much worse” to 5 “much better”; ^b^Combined estimate from 100 analyses based on imputed dataset. Abbreviations: AcroQoL, Acromegaly Quality of Life questionnaire; ATG, autogel; CI, confidence interval; ITT, intention-to-treat; LAR, long-acting repeatable; LS, least squares; PSS, Patient Satisfaction Scale; SoC, standard of care; TSQM, Treatment Satisfaction Questionnaire for Medication.

The impact of treatment on QoL and treatment/patient satisfaction was assessed using patient-reported outcomes (Fig. 5). CAM2029-treated patients experienced improvements in AcroQoL total score at week 24 vs SoC at baseline [LS mean change 4.69 (95% CI: 1.51, 7.86)], including both physical [3.97 (95% CI: 0.35, 7.59)] and psychological [5.05 (95% CI: 1.68, 8.42)] domains (Fig. 5). In the placebo arm, there were numerically smaller changes in AcroQoL total score [LS mean change 2.24 (95% CI: −2.25, 6.72)] and psychological domain [4.43 (95% CI: −0.31, 9.18)], while the physical domain worsened [−1.20 (95% CI: −6.35, 3.95)]. EQ-5D-5L visual analogue scale scores were similar at week 24 vs baseline [mean change CAM2029: 0.9 (95% CI: −2.4, 4.3); placebo: 0.1 (95% CI: −5.8, 6.0)].

TSQM convenience score increased at week 24 vs SoC at baseline (Fig. 5), with a numerically greater improvement in CAM2029-treated patients [LS mean change CAM2029: 13.85 (95% CI: 9.45, 18.25); placebo: 9.90 (95% CI: 4.06, 15.75)]. Change from baseline for other TSQM domains favored CAM2029 (Supplementary Table S4) (25).

The mean PSS score was 3.9 (95% CI: 3.6, 4.2) for CAM2029-treated patients and 3.4 (95% CI: 2.9, 3.8) for placebo-treated patients (Fig. 5); while formal statistical testing was not performed, there were more favorable responses in the CAM2029 group (Supplementary Fig. S2) (25). On the PSS, the proportion of patients who responded that CAM2029 was either “slightly better” or “much better” than their previous treatment was 56.0% (14/25) for those receiving octreotide LAR at baseline and 39.1% (9/23) for those receiving lanreotide ATG at baseline.

Most patients chose self-/partner-administration of treatment [CAM2029: 35/48 (72.9%); placebo: 22/24 (91.7%)]; 32/35 (91.4%) and 20/22 (90.9%) patients, respectively, were declared competent to do so. Overall, 29/48 (60.4%) CAM2029-treated and 19/24 (79.2%) placebo-treated patients self-administered or had their partner administer treatment at ≥1 visit. Per SIAQ scores, patients who self-administered reported improvements in “satisfaction with current way of taking medication” after first administration [mean change: CAM2029: 1.02 (95% CI: 0.28, 1.76); placebo: 1.03 (95% CI: −0.26, 2.32)] and from baseline SoC to week 20 [0.38 (95% CI: −0.84, 1.59) and 1.67 (95% CI: −0.99, 4.32), respectively]. Additional SIAQ domains are reported in Supplementary Table S4 (25).

Overall, 56/71 (78.9%) patients experienced ≥1 AE [CAM2029: 37/47 (78.7%); placebo: 19/24 (79.2%)], most of which were mild (Table 2). No SAEs were considered CAM2029-related; one treatment-related SAE (cholecystitis) was recorded on day 34 in the placebo arm. In the CAM2029 arm, 4/47 (8.5%) patients discontinued treatment due to AEs [injection site reaction (n = 3) and migraine (n = 1); Table 3]. In the placebo arm, 1/24 (1.4%) patients discontinued treatment due to injection site erythema (Table 3). No AEs led to dose reduction or trial withdrawal. Of all patients, 8/71 (11.3%) reported COVID-19-related AEs.

**Table 2.** Summary of AEs and incidence of AEs reported by ≥5% of patients in either treatment arm

n (%) of patients	CAM2029 n = 47	Placebo n = 24	Total n = 71
Any AE	37 (78.7)	19 (79.2)	56 (78.9)
Any treatment-related AE	24 (51.1)	15 (62.5)	39 (54.9)
Any grade 1 AE*^a^*	34 (72.3)	18 (75.0)	52 (73.2)
Any grade 2 AE*^a^*	19 (40.4)	8 (33.3)	27 (38.0)
Any grade 3 or higher AE*^a^*	5 (10.6)	2 (8.3)	7 (9.9)
Any SAE	4 (8.5)	2 (8.3)	6 (8.5)
Any treatment-related SAE*^b^*	0	1 (4.2)	1 (1.4)
Any AE leading to treatment discontinuation	4 (8.5)	1 (4.2)	5 (7.0)
Any AE leading to withdrawal from trial	0	0	0
Any AE leading to dose reduction	0	0	0
Any death	0	0	0
Any COVID-19-related AE	5 (10.6)	3 (12.5)	8 (11.3)
**Preferred term (unless stated)**			
Any injection site AE*^c^*	19 (40.4)	15 (62.5)	34 (47.9)
Arthralgia	8 (17.0)	2 (8.3)	10 (14.1)
COVID-19	4 (8.5)	3 (12.5)	7 (9.9)
Fatigue	3 (6.4)	1 (4.2)	4 (5.6)
Pruritus	4 (8.5)	0	4 (5.6)
Anemia	1 (2.1)	3 (12.5)	4 (5.6)
Abdominal pain upper	3 (6.4)	0	3 (4.2)
Headache	3 (6.4)	0	3 (4.2)
Leukopenia	0	2 (8.3)	2 (2.8)

Safety analysis set. AEs were coded using MedDRA version 25.0.

Abbreviations: AE, adverse event; CTCAE, Common Terminology Criteria for Adverse Events; NCI, National Cancer Institute; MedDRA, Medical Dictionary for Regulatory Activities; SAE, serious adverse event; SOC, system organ class.

^*a*^Severity assessed according to the NCI CTCAE v5.0. The severities of mild, moderate, and severe correspond to Grades 1 to 3. AEs considered life-threatening, resulting in death, requiring inpatient hospitalization or prolongation of existing hospitalization, resulting in persistent or significant disability/incapacity, consisting of a congenital anomaly or birth defect, or serious medically important events as judged by the investigator, were not graded but were considered SAEs.

^*b*^SAEs assessed as possibly or probably related to treatment.

^*c*^All injection site AEs belong to the SOC General disorders and administration site conditions.

**Table 3.** Summary of treatment-related SAEs and AEs leading to treatment discontinuation

n of patients	CAM2029 n = 47	Placebo n = 24	Total n = 71
Treatment-related SAEs*^a^*			
Cholecystitis	0	1	1
AEs leading to treatment discontinuation			
Injection site induration	2	0	2
Injection site erythema	1	1	2
Migraine	1	0	1

Safety analysis set. AEs were coded using MedDRA version 25.0. AEs constituted SAEs if they were considered life-threatening, resulted in death, required inpatient hospitalization or prolongation of existing hospitalization, resulted in persistent or significant disability/incapacity, consisted of a congenital anomaly or birth defect, or were considered medically important events as judged by the investigator.

Abbreviations: AE, adverse event; MedDRA, Medical Dictionary for Regulatory Activities; SAE, serious adverse event.

^*a*^SAEs assessed as possibly or probably related to treatment.

Injection site reactions were the most common type of AE, reported in a total of 34/71 (47.9%) patients, comprising 19/47 (40.4%) CAM2029-treated patients and 15/24 (62.5%) placebo-treated patients. All injection site AEs were mild or moderate and none were severe; the majority were transient and lasted <2 months, with decreasing incidence rates over time. The most common was injection site reaction, and the most common AE overall was injection site erythema [CAM2029: 12/47 (25.5%); placebo: 5/24 (20.8%)]. Injection site pain, reported on a 0 to 10 scale, was comparable between arms, with an overall mean of 2.1 (SD: 1.8) immediately after injection and 0.3 (SD: 0.7) after 1 hour. No patients in the CAM2029 arm had corrected QT interval >450 ms, vs 1/24 (4.2%) in the placebo arm (>450-≤480 ms). No new or unexpected safety signals were identified.

## Discussion

In this phase 3 trial that included patients who had been receiving stable SoC treatment and had biochemically controlled acromegaly at screening, those who received CAM2029 demonstrated significantly greater biochemical disease control, measured as IGF-1 and GH over 24 weeks, compared to placebo. IGF-1 is considered the most reliable biomarker of disease activity for patients with acromegaly and the dominant driver for treatment decisions (3, 4). Sensitivity and supportive analyses confirmed the superiority of CAM2029 over placebo for the primary and key secondary outcomes. No new or unexpected safety findings for CAM2029 were observed, and the safety profile was consistent with SoC, despite higher octreotide exposure compared to octreotide LAR (38, 39).

The significant difference between CAM2029 and placebo in IGF-1 response rate, and the change from baseline in IGF-1/ULN, was supported by individual patient data, with all but 1 patient in the CAM2029 arm maintaining stable IGF-1 values below or slightly above the ULN at baseline and end of trial, while most patients in the placebo arm demonstrated increasing IGF-1, such that they were slightly or substantially above ULN at end of trial.

Over 70% of all CAM2029-treated patients had IGF-1 ≤ULN (threshold for biochemical control) at week 22/24, including those who had already lost biochemical control by baseline (8.3% in each arm; CAM2029: 4/48; placebo: 2/24) and those who discontinued treatment prior to week 22 who were classified as nonresponders in the primary and secondary analyses irrespective of IGF-1 status at their final assessment. In contrast, less than 40% of placebo-treated patients maintained the biochemical control that they had at screening. Of those patients in the CAM2029 arm who were classified as nonresponders for the primary endpoint, all had IGF-1 values <1.3 × ULN (threshold for diagnosis of acromegaly) at the end of the trial, except for 1 patient with a single high IGF-1 value at week 22 (which subsequently decreased to <ULN at week 24). Expected variability in the bioanalytic results may have affected the outcomes, especially for patients whose IGF-1 values were equal to or only slightly below ULN at screening. Intraindividual, week-to-week variation in IGF-1 levels has been reported to impact the assessment of biochemical control in acromegaly patients (40). However, the IGF-1 and GH concentrations in serum samples were analyzed in a central laboratory using validated assays, ensuring precision and accuracy of measurements.

Thus, the loss of biochemical control in a small proportion of CAM2029-treated patients was not unexpected, while the placebo response rate was anticipated and accounted for in the power analysis. Indeed, the placebo response rate likely reflects the stability of enrolled patients, their long treatment histories (mean SoC treatment time at baseline was 7.7 years), and the relatively short trial duration utilized to minimize the unnecessary use of placebo. Previous studies have reported a significant proportion of patients with acromegaly continue to have biochemical control of disease after discontinuing SoC treatment for up to 12 months, as well as significant control of GH levels for patients switching from stable SoC treatment to placebo (29, 37, 41). Nevertheless, at the individual patient level, most patients in the placebo arm demonstrated increasing IGF-1 values from baseline to end of trial, such that some patients receiving placebo who were responders in the primary and secondary analyses would likely have lost biochemical control if they continued to receive placebo for a longer duration. In contrast, almost all patients in the CAM2029 arm had IGF-1 values at the end of trial that were similar or lower than baseline, including a number of nonresponders with IGF-1 only slightly above ULN at end of trial. Additionally, all CAM2029-treated patients who discontinued treatment, and were thus considered nonresponders, still had IGF-1 values <ULN with biochemical control of disease at the time of discontinuation. Furthermore, while a GH threshold of 2.5 μg/L was employed for the second key secondary endpoint, in accordance with guidance from the US Food and Drug Administration, in practice mean GH remained ≤1.0 μg/L in CAM2029-treated patients throughout the duration of the trial, in line with current guidelines for the treatment of acromegaly (7).

While biochemical control is the main objective of acromegaly treatment (3, 42), patients with biochemical control often report persistent disease symptoms, and there is a discordance between clinically reported outcomes and patient-reported severity of acromegaly symptoms (19, 21). Unlike placebo-treated patients who had mean AIS Overall Scores that were similar to baseline SoC throughout the trial, mean AIS Overall Scores were consistently numerically lower than baseline SoC, and numerically lower than placebo, in patients treated with CAM2029. This indicates that, in addition to biochemical control, CAM2029 was effective at controlling symptoms and lowering the severity of disease experienced by patients in the trial.

Existing SRL treatments often do not result in meaningful QoL improvements for patients with acromegaly due to the treatment burden on daily life and work (20). Thus, there is a growing focus on improved QoL as a key treatment goal (18), and more convenient therapeutic options that can achieve this by lowering treatment burden would be highly valuable. While home injection of SRLs is considered safe, associated with high treatment adherence and satisfaction, and approved for use in some countries (8, 43), there are still barriers to home injection using current treatments and no long-acting SRL is currently indicated for self- or partner injection in the United States. Indeed, evidence suggests that few patients receive SRL injections at home, and fewer still self-administer injections (44); this is likely influenced by lanreotide ATG requiring deep subcutaneous injection in the upper buttocks using a larger needle (11). Studies of nurse and patient experiences of SRLs currently approved for self-injection have focused solely on healthcare provider-administered injections or have found that only a minority of patients are self-injecting (10, 45, 46). CAM2029 was designed to improve treatment satisfaction by facilitating convenient dosing and easy self-administration, combined with a higher gauge needle than SoC (22G vs 18G) (22).

In the present study, most patients/partners were deemed competent to self-administer with clinical supervision. While treatment satisfaction improvements from baseline were observed in both arms, changes were more favorable in CAM2029-treated patients. Patients commented that autonomy of self-administration and ease of injection were contributory. Indeed, while improvements in the AcroQoL psychological domain occurred in both arms, likely reflecting increased autonomy and convenience, physical domain improvements only occurred in CAM2029-treated patients. Collectively, these data suggest that CAM2029 is a treatment option that is both effective and convenient, contributing to a more favorable overall treatment acceptability profile and improved QoL than current SoC, alongside safety that is consistent with SoC.

This trial had some limitations, including that it coincided with major world events, such as the COVID-19 pandemic, affecting patient recruitment and some patients’ visit schedules (Supplementary Material S3) (25). Six patients in the CAM2029 arm and 1 patient in the placebo arm had protocol deviations whereby >35 days elapsed between the last treatment dose and the final sample being taken for IGF-1 measurement; 3 of these were due to COVID-19-related disruptions. These protocol deviations may have further contributed to variability in the bioanalytic results. Nevertheless, IGF-1 remained well controlled in these patients.

Acromegaly is a rare disease with a low global population size (1), thus resulting in a modest sample of patients being recruited for this trial. Nonetheless, the sample size was sufficient to meet the trial's stringent endpoint criteria. While the relatively short duration of this trial precluded long-term assessment of CAM2029 benefit, patients could subsequently enroll in a long-term open-label trial (ACROINNOVA 2; NCT04125836) including an expanded patient population with IGF-1 >ULN and ≤2 × ULN, that is underway to further assess safety, biochemical control, signs/symptoms, QoL, and treatment satisfaction with CAM2029.

The placebo control arm was a regulatory requirement for this trial. While a head-to-head design would be necessary to formally compare CAM2029 with SoC treatment, CAM2029 did demonstrate substantial improvements in QoL and treatment satisfaction compared to SoC at baseline, despite patients already being biochemically controlled at screening and on stable long-term treatment (octreotide LAR or lanreotide ATG) at variable doses. These improvements were numerically greater than those in placebo-treated patients. Importantly, CAM2029-treated patients did not experience a worsening of clinical signs and symptoms, with AIS scores consistently lower than baseline SoC. Our findings suggest that a direct shift to monthly CAM2029 20 mg, irrespective of prior treatment and dose, can reduce patients’ treatment burden compared to SoC, while providing continued, or improved, biochemical and symptom control. The results from the ongoing open-label trial will further elucidate the long-term efficacy and safety of CAM2029.

The present double-blind, placebo-controlled trial demonstrated the superior efficacy of CAM2029 for biochemical control of acromegaly compared with placebo, alongside improvement in the AcroQoL physical domain and treatment satisfaction compared to SoC, while safety was consistent with SoC. These findings support the promising potential of CAM2029 to provide an effective and convenient treatment option for acromegaly that improves patient QoL.

## Data Availability

Restrictions apply to the availability of some or all data generated or analyzed during this study to preserve patient confidentiality or because they were used under license. The corresponding author will on request detail the restrictions and any conditions under which access to some data may be provided.
